# Cypriot Healthcare Professionals’ Knowledge and Skills When Interacting With Patients With Aphasia in Clinical Settings

**DOI:** 10.1111/1460-6984.70089

**Published:** 2025-07-03

**Authors:** Marina Charalambous, Phivos Phylactou, Marios Serafim, Pinelopi Vlotinou, Anastasios M Georgiou, Eliada Pampoulou, Maria Papaioannou, Fotini Georgiou, Lakis Palazis

**Affiliations:** ^1^ Department of Rehabilitation Sciences, School of Health Sciences Cyprus University of Technology Limassol Cyprus; ^2^ School of Physical Therapy University of Western Ontario London Canada; ^3^ The Gray Centre for Mobility and Activity, Parkwood Institute London Canada; ^4^ Department of Neurology, School of Medicine Democritus University of Thrace Alexandroupolis Greece; ^5^ Department of Occupational Therapy University of West Attica Athens Greece; ^6^ Intensive Care Unit, Limassol General Hospital, State Health Services Organisation Limassol Cyprus

**Keywords:** aphasia, health professionals and aphasia questionnaire, healthcare professionals, HPAQ, speech and language therapy

## Abstract

**Background:**

Aphasia, a communication disorder mainly resulting from stroke, poses challenges to the meaningful interactions between healthcare professionals and people with aphasia (PWA). Little is known about the knowledge and skills of Cypriot healthcare professionals when interacting with PWA in clinical settings. This study explores the knowledge and skills of Cypriot healthcare professionals in communicating with PWA.

**Method:**

A total of 245 Greek‐speaking Cypriot healthcare professionals completed the Greek version of the Health Professionals and Aphasia Questionnaire (HPAQ). Participants’ knowledge and skills were assessed in five categories: Knowledge, Skills, Behaviour and Emotions, Practice, and Environment. Demographic data were analysed through linear regression and ANOVA to identify predictors of HPAQ scores.

**Results:**

Participants’ mean HPAQ score was 94.57 (SD = 28.1). Higher educational levels (master's degree) and the Speech and Language Therapy profession significantly predicted higher HPAQ scores. Moderate frequency of interaction with PWA (1–20 times/week) was also associated with improved knowledge of aphasia and better communication skills when interacting with PWA.

**Conclusion:**

Speech and Language Therapists exhibited higher competency in interacting with PWA compared to other healthcare professionals, highlighting their specialised training. The findings emphasise the importance of education, experience, and interaction frequency in enhancing healthcare professionals' ability to communicate effectively with PWA in clinical settings. To address identified gaps, targeted interventions, such as Communication Partner Training, are recommended to improve communication strategies and patient outcomes in Cypriot healthcare settings.

**WHAT THIS PAPER ADDS:**

*What is already known on this subject*

- Aphasia, which affects 40% of stroke survivors in the acute phase, profoundly impacts communication abilities, leading to longer hospital stays, increased healthcare needs, and higher rates of disability. Many healthcare professionals lack the training, strategies, and resources required to effectively communicate with people with aphasia (PWA), limiting PWA's participation in decision‐making and negatively impacting the quality of care. In Cyprus, there is an urgent need to evaluate and enhance healthcare professionals' competencies in supporting PWA, highlighting the importance of communication training programs in improving care quality and patient outcomes.

*What this paper adds to the existing knowledge*

- This research highlights the critical need for accessible training programs at all educational levels to ensure equitable standards of stroke care in Cyprus. Moderate interaction with PWA plays a pivotal role in improving healthcare professionals' communication skills, emphasising the importance of balanced and sustainable contact opportunities that foster professional development while minimising the risk of burnout. The findings also reveal that professional experience alone does not enhance communication outcomes unless accompanied by purposeful training and meaningful interaction with PWA. The study underscores the need for targeted communication training for healthcare professionals to improve interdisciplinary collaboration and patient care outcomes.

*What are the potential or actual clinical implications of this work?*

- The clinical implications are as follows (a) there is a critical need to implement Communication Partner Training programs in Cypriot healthcare settings to address knowledge and skills gaps among non‐speech language therapy (SLT) healthcare professionals; (b) communication training programs can create more inclusive clinical environments, enabling PWA to actively participate in healthcare decisions and rehabilitation planning; (c) training interventions should be tailored to different professional groups and educational levels, with introductory‐level modules for non‐SLT professionals and advanced modules for SLTs; (d) communication training has the potential to improve the quality of care, patient satisfaction, and rehabilitation outcomes for PWA; (e) these actions will provide a basis for developing scalable and targeted communication training solutions to meet diverse healthcare needs in Cyprus.

## 1 Introduction

Aphasia is an acquired communication disorder mainly resulting from stroke (Cichon et al. 2021). Depending on the severity of brain damage and the location of the lesion, aphasia can impact all aspects of communication, including speech, comprehension, reading, and writing (Sheppard and Sebastian 2021). It occurs in 40% of stroke survivors during the acute phase (up to 3 weeks after the event, while they are hospitalised), with 30% continuing to experience persistent aphasia in the subacute (up to 3 months post‐stroke) phase (Mitchell et al. 2020). Aphasia is associated with prolonged hospital stays (Wu et al. 2020), higher rates of permanent disability (Levy et al. 2024), and significant barriers to accessing and participating in rehabilitation, exacerbated by recruitment and retention challenges (Georgiou and Kambanaros 2023).

People with aphasia (PWA) report negative experiences with healthcare interactions when in the hospital (Anemaat et al. 2024; Lamborn et al. 2024). This is related to self‐reported neglect from healthcare professionals (Isaksen et al. 2023) due to minimal time devoted to them (Loft et al. 2022), healthcare professionals dominating discussions (Bright and Reeves 2022), and limited use of communication strategies during interactions (van Rijssen et al. 2022). Consequently, PWA have reduced participation in health and rehabilitation‐related decision‐making (Charalambous et al. 2022; Raven‐Takken et al. 2024), resulting in lower‐quality care (Lamborn et al. 2024), often jeopardising their safety in the clinical settings (Burgener 2020) and rehabilitation outcomes (Gopaul et al. 2023).

Clinical settings' characteristics significantly foster, enhance, or hinder effective communication between healthcare professionals and PWA (Bright and Reeves 2022). Clinical settings hosting huge numbers of patients, like tertiary hospitals, generate significant barriers to individualised patient management by healthcare professionals due to time restrictions and limited access to resources and strategies (Carragher et al. 2024). The severity of the aphasic symptoms poses a significant challenge for healthcare professionals (Söderhielm et al. 2023) as they often lack knowledge and training in such communication disorders (Hur and Kang 2022). The persisting expressive and receptive language difficulties in PWA often surpass the collective efforts of healthcare professionals to establish contact with them (Hur and Kang 2022). The study by Cameron et al. (2018) found that health professionals without speech‐language pathology training had significantly lower confidence and identified fewer communication strategies when interacting with PWA, highlighting the need for formal training to improve their communication skills and support patient‐centred care. Also, the limited knowledge and access to augmentative or alternative communication systems (Huang et al. 2024; Jansson et al. 2019), and inadequate information about stroke and aphasia (Demers et al. 2025) hinder healthcare professionals’ functional and effective communication with PWA (Carragher et al. 2024).

Healthcare professionals view communication difficulties as the primary reason for the limited involvement of PWA in goal‐setting and shared decision‐making during the planning of therapeutic interventions (Söderhielm et al. 2023). This includes misinterpretations and misunderstandings of medical instructions and interventions (Anemaat et al. 2024). Consequently, the choices and decisions made by healthcare professionals may not align with the pragmatic needs of PWA (Hinckley and Jayes 2023). With adequate knowledge and skills, healthcare professionals can enhance access to the most appropriate therapeutic interventions for PWA (Jensen et al. 2022). Furthermore, learning compensatory communication skills and strategies effectively improves communication with PWA, resulting in better healthcare delivery (Carragher et al. 2024), rehabilitation outcomes, and satisfaction with healthcare services (van Rijssen et al. 2022). Additionally, it positively influences patient safety and the quality of healthcare services (Burgener 2020). It also encourages the participation of PWA in healthcare activities and shared decision‐making (Bright and Reeves 2022) and plays a crucial role in establishing personalised rehabilitation goals (Hinckley and Jayes 2023). This is especially important since the World Stroke Organisation Global Stroke Services Guidelines and Action Plan (Lindsay et al. 2014) stress the necessity of supporting stroke patients in healthcare decision‐making and individualised goal‐setting. Therefore, training healthcare professionals in communication enhancement programs such as Communication Partner Training is vital for improving patient outcomes in hospitals (Sullivan et al. 2024).

Communication Partner Training refers to the interventions designed to improve the communication between PWA and their communication partners (Simmons‐Mackie et al. 2016). This training focuses on understanding aphasia and adopting communication strategies (Jensen et al. 2022). Communication Partner Training interventions are implemented through various methods, including training in communication strategies, providing feedback, and practicing skills either individually or in groups (Isaksen et al. 2023). Communication Partner Training is typically facilitated by Speech and Language Therapists (SLTs), and it's directed towards people with communication impairments, family members, caregivers, and healthcare professionals (Fitzmaurice et al. 2024). Implementing Communication Partner Training in healthcare environments has been shown to increase awareness and knowledge of aphasia, improve communication and conversational skills among healthcare professionals, and enhance their ability to support PWA (Cruice et al. 2018). However, despite the extensive global evidence supporting CPT, there is a notable lack of research and implementation in Cyprus, and this study aims to address this gap. In this study, we refer both to general education and training on communicating with PWA, as well as to CPT as a specific, evidence‐based intervention. CPT is proposed as one potential implementation pathway that can build on the training needs identified through this research.

This study aims to investigate (1) how Cypriot healthcare professionals rate their knowledge and skills in communicating with PWA, and (2) whether profession, education level, and frequency of interaction with PWA predict these self‐assessments. This study holds significant importance in Cyprus, as it addresses a critical gap in measuring the knowledge and skills of healthcare professionals when interacting with PWA in clinical settings. It aspires to create a foundation for implementing Communication Partner Training programs in Cypriot healthcare settings to enhance the quality of care and rehabilitation outcomes for PWA. Tailored strategies, alongside education and awareness‐raising efforts, have the potential to enhance healthcare professionals’ communication skills, contribute to a more inclusive and supportive environment for PWA, support patient participation in healthcare decisions, and foster more effective clinical interactions.

## 2 Methods

Approval for the conduct of the research project was obtained (CNBC EP 2023.01.02) before the initiation of the study.

### 2.1 Materials

#### 2.1.1 The Health Professionals and Aphasia Questionnaire (HPAQ)

The Health Professionals and Aphasia Questionnaire (HPAQ) is a self‐reported questionnaire that measures the knowledge, skills, and confidence of healthcare professionals regarding communication with PWA (Jensen et al. 2022). The HPAQ was developed to measure the outcomes of Communication Partner Training in intervention studies (Jensen et al. 2022). The tool was developed from two pre‐existing self‐report tools: (1) the Communicative Access Measure for Stroke for Frontline Practice (CAMS2; Kagan et al. 2017), which includes questions to evaluate the accessibility of PWA (at the institutional level, frontline staff level, and patient satisfaction level) and assess healthcare professionals’ knowledge and perception about aphasia and (2) the simplified version of the Knowledge of Aphasia Questionnaire (KAQ; Simmons‐Mackie et al. 2007), a self‐report tool designed to assess healthcare professionals’ knowledge, behaviour, and attitudes towards PWA.

The HPAQ consists of 16 questions distributed across five categories: (i) Knowledge (basic information about aphasia and communication), (ii) Skills (communication strategies), (iii) Attitudes and Emotions (feelings associated with the inability to express oneself), (iv) Practice (implementation of communication skills), and (v) Environment (supportive or non‐supportive role of the work) (Jensen et al. 2022).

### 2.2 Design

A descriptive cross‐sectional study was carried out to assess the knowledge and skills of Cypriot healthcare professionals in communicating and interacting with PWA in clinical settings.

### 2.3 Participation Criteria

Participants were eligible to participate in the study if they were healthcare professionals actively working in clinical settings relevant to stroke care and rehabilitation, such as hospitals, private practices, rehabilitation centres etc., located in the five major healthcare regions of the country: Nicosia, Limassol, Paphos, Larnaca and Famagusta. Participants were excluded if they were not practicing in a setting that supports stroke care and rehabilitation.

### 2.4 Recruitment

Participants were recruited from various clinical settings in Cyprus. Recruitment sources included Nicosia Tertiary General Hospital, Limassol, Paphos, Larnaca and Famagusta Public Hospitals, as well as several private hospitals around the island and rehabilitation centres. Participants were identified through professional associations, hospital networks, and by distributing targeted digital invitations via email and online group messaging platforms.

### 2.5 Procedures

The translation and adaptation of the HPAQ into Greek was conducted by Dr. Efstratiadou and her team in 2023 at the University of Peloponnese. Written approval for the use of the HPAQ was received from Dr. Jytte Isaksen and Dr. Efstratiadou. Greek‐speaking healthcare professionals from Cyprus were invited to complete the Greek version of the HPAQ between March and June 2024. Participants were informed in writing about the purpose of the study, the content of the questionnaire, and how their data would be managed. The completion of the questionnaire was anonymous, and each participant provided written consent before the completion of the questionnaire.

The HPAQ was completed by healthcare professionals in two formats: a printed hardcopy and an electronic form (Google Forms). The electronic version was identical to the printed, and the completion time was approximately 10 min. Participants were given the option to complete either a printed or electronic version of the questionnaire, based on their personal preference and convenience. This dual‐format approach aimed to improve accessibility across diverse clinical settings and accommodate varying levels of digital literacy. For each question, a Likert scale from 1 to 10 was provided, where each healthcare professional marked a number to indicate what corresponds to themselves, with zero representing no knowledge/strong disagreement and ten representing excellent knowledge/strong agreement. While completing the questionnaire, healthcare professionals provided demographic information based on their work context.

### 2.6 Data Analysis

Statistical analysis was conducted using JASP (v0.19.1; JASP Team 2024). To identify the predictors of the HPAQ scores, a linear regression model was applied. This model included gender, education level, profession, work environment, years of professional experience, years of experience with individuals with aphasia following a stroke, and frequency of working with individuals with aphasia following a stroke as predictors. To further investigate potential group differences between the predictor factors and the participants’ scores on the HPAQ, ANOVA analyses were performed for each factor separately. For the linear regression and for each ANOVA, Q–Q plots were examined to ensure that residuals were normally distributed, and a Levene's test was performed to test for between group variance homogeneity. The residuals were normally distributed around 0 for all tests. ANOVAs that violated homogeneity were meant to be adjusted through a Welch correction, though a correction was not deemed necessary (Levene's: all *Fs* < 2, all *ps* > 0.101). Statistical significance was determined with an *α* = 0.05.

## 3 RESULTS

### 3.1 Participants

A total of 245 healthcare professionals participated in the study. The majority were female (76%), with most aged between 26 and 35 years (mean = 94.9, SD = 28.02). Table 1 provides the demographic characteristics of the participants.

**TABLE 1:** Demographic characteristics of participants.

Domain	Count (*%*)	Mean HPAQ score (*sd*)
*Sex*		
Female	187 (76%)	96.48 (26.79)
Male	58 (24%)	88.4 (31.43)
*Age group*		
18–25	51 (21%)	97.71 (26.16)
26–35	105 (43%)	94.9 (28.02)
36–45	46 (19%)	88.17 (32.07)
46–55	26 (11%)	101.73 (22.21)
56–65	16 (6%)	87.81 (29.83)
N/K	1 (<1%)	–
*Education level*		
Middle school (Grade 9)	2 (<1%)	124 (26.87)
High school (Grade 12)	10 (4%)	85.5 (17.6)
College	2 (<1%)	81.5 (34.65)
Bachelor's	140 (57%)	90.36 (26.41)
Master's	87 (36%)	102.17 (30.49)
Doctorate	4 (2%)	91 (13.9)
*Work location*		
Famagusta	3 (1%)	126.33 (15.31)
Larnaca	4 (2%)	115.75 (24.07)
Limassol	190 (78%)	93.88 (28.06)
Nicosia	39 (16%)	110.9 (19.54)
Paphos	9 (3%)	92 (38.96)
*Profession*		
Nurse assistant	19 (8%)	92.53 (25.4)
Nurse	131 (53%)	90.56 (27.53)
Physician	27 (11%)	81.07 (25.35)
Speech & language therapist	43 (18%)	116.60 (22.28)
Rehabilitation expert ^a^(OT/PT/Psy)	25 (10%)	93.08 (26.94)
*Work setting*		
Adult day centre	3 (1%)	77 (17.09)
Home‐based care	7 (3%)	99 (32.8)
Nursing home	2 (1%)	115.5 (40.31)
Private hospital	112 (46%)	95.42 (28.82)
Private practice	22 (10%)	114.90 (20.33)
Public hospital	78 (32%)	86.54 (24.11)
Rehabilitation centre	19 (8%)	104.21 (28.1)
Clinical placement	1 (<1%)	61 (N/A)
N/k	1 (<1%)	–
*Years of work experience*		
0–5	102 (42%)	96.35 (27.23)
6–10	46 (19%)	88.37 (30.75)
11–15	35 (14%)	88.03 (29.96)
16–20	24 (10%)	103.83 (26.73)
>21	37 (15%)	97.24 (24.88)
*Years of work experience with people with aphasia*		
0–5	148 (60%)	92.19 (29.02)
6–10	30 (12%)	101.8 (26.87)
11–15	24 (10%)	94.25 (31.25)
16–20	19 (8%)	100.63 (24.41)
>21	23 (9%)	95.3 (22.89)
N/K	1 (<1%)	–
*Frequency with people with aphasia (times per week)*		
0	49 (20%)	81.49 (33.42)
1–10	164 (67%)	96.9 (25.75)
11–20	24 (10%)	101 (24.97)
21–30	4 (2%)	104 (32.83)
>31	3 (1%)	119.33 (14.36)
N/K	1 (<1%)	–

Abbreviations: N/K, not known; OT, occupational therapist; Psy, psychologist; PT, physical therapist.

^a^OT count: 5 (2%), HPAQ: 73.20 (35.68); PT count: 19 (8%), HPAQ: 96.37 (22.09); Psy count: 1 (<1%), HPAQ: 130.

### 3.2 HPAQ Score Predictors

**FIGURE 1:**
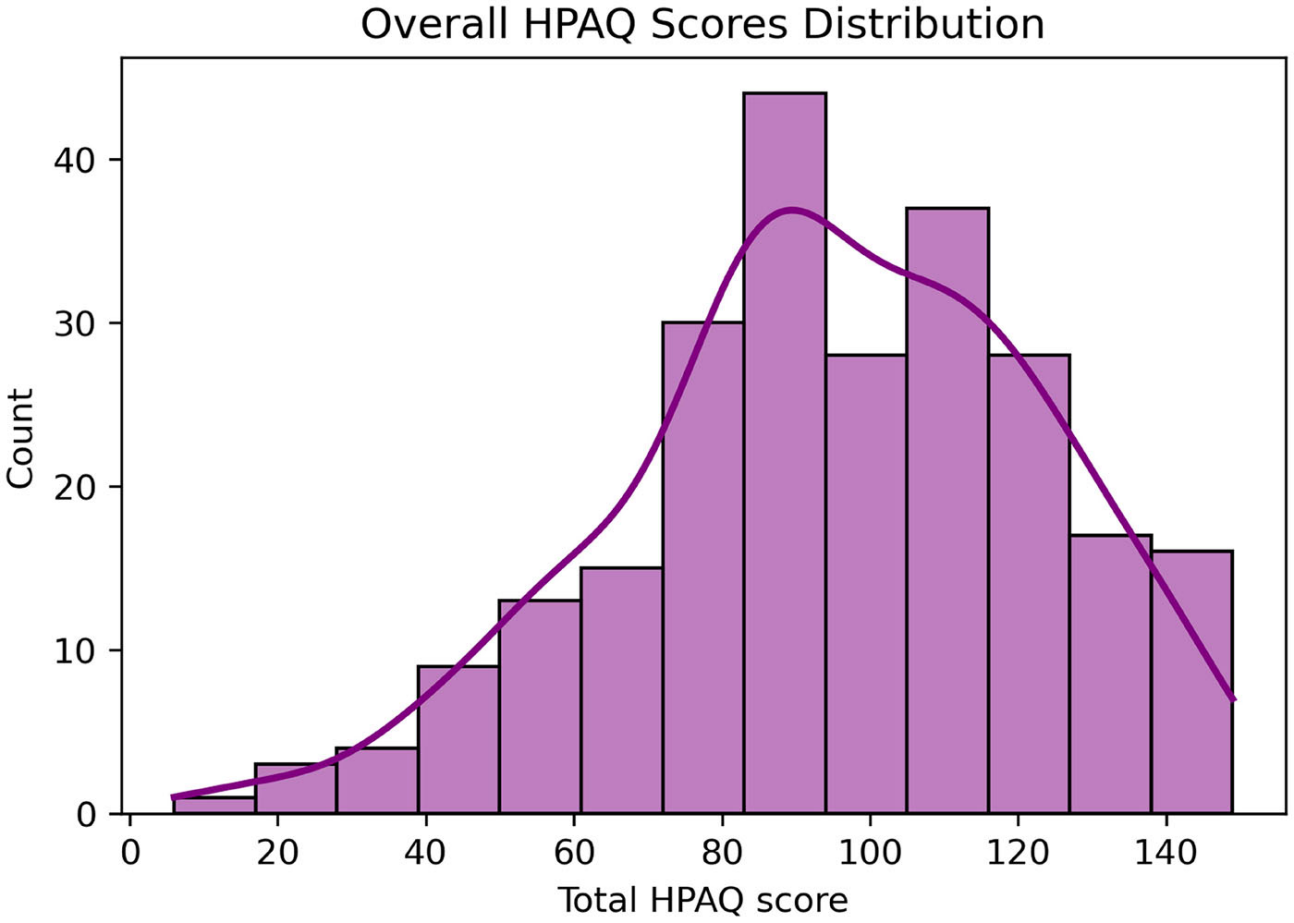
Overall distribution of HPAQ scores from 245 participants in Cyprus. HPAQ, health professionals and aphasia questionnaire.

Overall, the participants’ mean HPAQ score was 94.57 (*sd* = 28.1, [*min* = 6, *max* = 149]). The distribution of the HPAQ scores is depicted in Figure 1 (for subgroup HPAQ scores see Table 1).

To identify the factors predicting the HPAQ scores, we implemented a linear regression model including sex, education level, profession, work setting, years of work experience, years of work experience with PWA, and frequency of working with PWA, as predictive factors. The model provided a decent fit (*R^2^_adj_* = 0.302) and yielded a significant result (*F_(34,208)_* = 4.073, *p* < 0.001). The significant predictors that were identified included educational level (high‐school grade 9, *t* = 2.011, *p* = 0.046; Master's, *t =* 2.144, *p* = 0.033), profession (SLT, *t =* 2.354, *p* = 0.02), years of work experience (>21, *t* = 2.025, *p* = 0.044), years of work experience with PWA (6–10, *t* = 2.255, *p* = 0.025), and frequency of working with PWA (1–10, *t* = 3.561, *p* < 0.001; 11–20, *t* = 1.993, *p* = 0.048; 21–30, *t* = 2.268, *p* = 0.024). No significant results were evident for sex or work setting.

To further explore between group differences for each factor and the participants’ score in the HPAQ, we performed separate ANOVA analyses for each factor. These analyses are described in the following sections.

#### 3.2.1 Educational Level Differences

**FIGURE 2:**
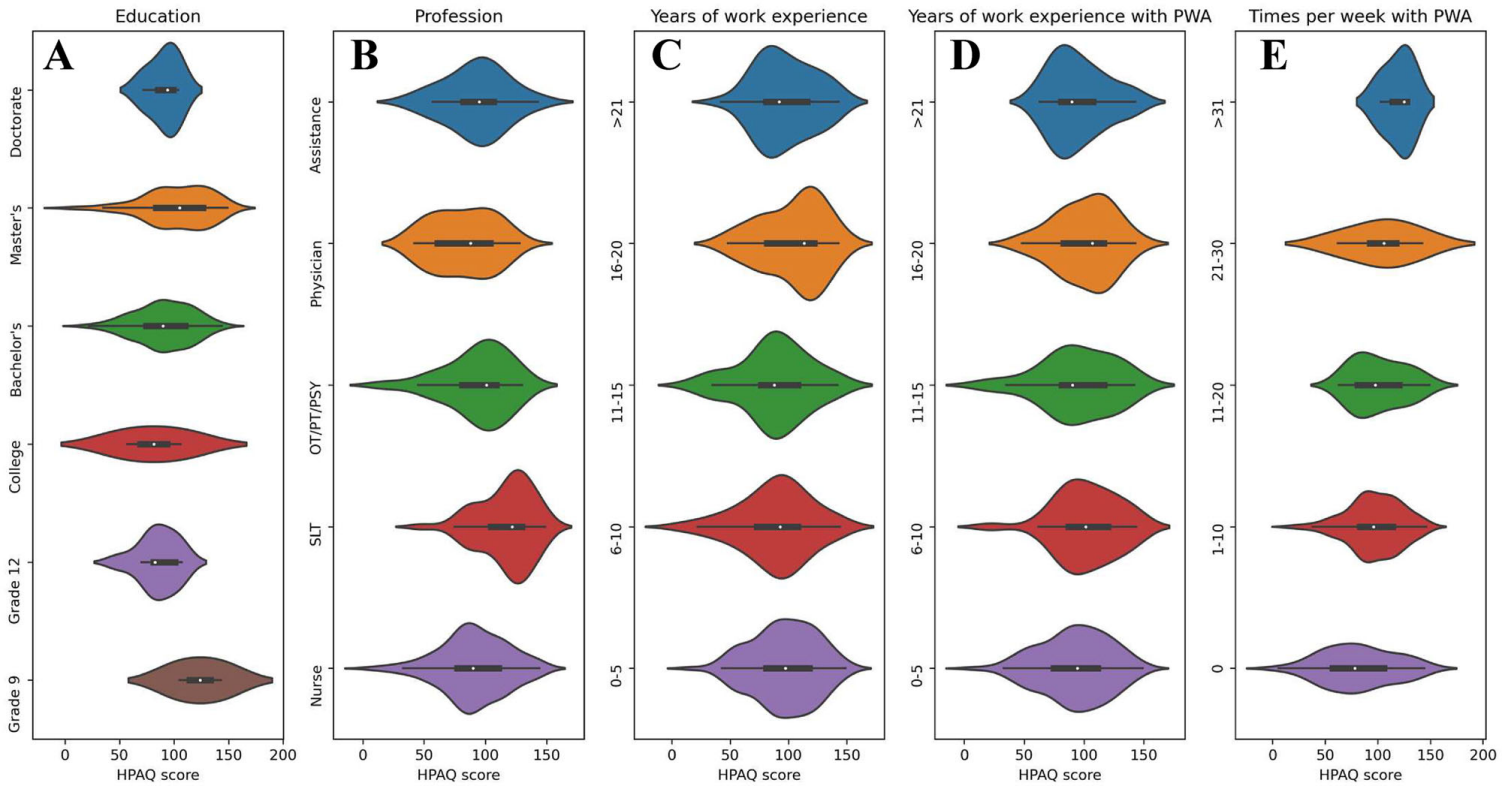
Subgroup HPAQ scores across various factors, namely (A) Education, (B) Profession, (C) years of work experience, (D) yeas of work experience with PWA specifically, and (E) frequency of work with PWA, expressed as times per week. HPAQ, health professionals and aphasia questionnaire; PWA, people with aphasia.

The results of the ANOVA on education replicated the regression findings, indicating a significant effect of education level on HPAQ scores (*F_(5,239)_ =* 2.74, *p* = 0.02, partial *η* ^2^ = 0.05; Figure 2A). Tuckey corrected post‐hoc *t*‐tests, revealed that this difference was mainly driven by the higher HPAQ scores of participants with a master's degree, compared to those with a bachelor's degree (*t_(239)_* = 3.13, *p_tuckey_* = 0.02). This finding illustrates that additional years of education may increase knowledge and skills, though, given the small sample size for some of the subgroups, this result should be taken with caution.

#### 3.2.2 Profession Differences

The ANOVA on the profession subgroup echoed the results of the regression model, providing a significant difference on HPAQ scores based on profession (*F_(4,240)_ =* 10.02, *p* < 0.001, partial *η* ^2^ = 0.14; Figure 2B). In detail, this difference was driven by the higher HPAQ scores by SLTs, compared to all other profession subgroups (assistants: *t_(240)_* = 3.33, *p_tuckey_* < 0.001; nurses: *t_(240)_* = 26.04, *p_tuckey_* < 0.001; physicians: *t_(240)_* = 34.90, *p_tuckey_* < 0.001; other rehabilitation experts: *t_(240)_* = 23.52, *p_tuckey_* < 0.001). These results show that, overall, SLTs report higher knowledge and skills compared to other professionals working with PWA.

#### 3.2.3 Years of Work Experience Differences

Contrary to the results of the regression model, the ANOVA on the different subgroups based on years of work experience, did not reach significance (*F_(4,239)_ =* 1.89, *p* = 0.11, partial *η* ^2^ = 0.03; Figure 2C). Considering the significant effect of Years of Work Experience as a predictor in the regression model, and the lack of significant effects in the ANOVA, the effect of years of work experience on knowledge and skills related to PWA remains inconclusive.

#### 3.2.4 Years of Work Experience With PWA Differences

Years of work experience with PWA also failed to show any significant differences in the ANOVA of HPAQ scores (*F_(4,239)_ =* 0.98, *p* = 0.42, partial *η* ^2^ = 0.02; Figure 2D). Considering this, no strong conclusions can be drawn about how years of experience with PWA may relate to knowledge and skills related to PWA. Though, it is possible that the effect of years of experience with PWA on HPAQ scores, shown in the regression analysis, is driven by an underlaying interaction with another factor. Similarly to the previous subsection, further exploratory analyses of these potential interactions were not possible, due to the small number of observations in some of the interactions.

#### 3.2.5 Frequency of Working With PWA Differences

A significant ANOVA indicated that differences exist between the subgroups defined according to the frequency of working with PWA (*F_(4,239)_ =* 4.30, *p* < 0.001, partial *η* ^2^ = 0.07; Figure 2E). According to the Tuckey corrected post‐hoc *t*‐tests, these differences were driven by the lower HPAQ scores of healthcare professionals not working at all with PWA (0 times per week), compared to those who work with PWA 1–10 times per week (*t_(239)_* = 3.53, *p_tuckey_* < 0.001), and 11–20 times per week (*t_(239)_* = 2.91, *p_tuckey_* = 0.03). These findings show that a moderate, but not an excessive (i.e., <21 times per week) frequency of work with PWA may potentially lead to higher knowledge and skills related to PWA.

#### 3.2.6 Findings Summary

Overall, our results show that HPAQ scores are higher for healthcare professionals with higher (Master's) compared to lower education (bachelor's degree). Further, our findings suggest that SLTs have higher knowledge and skills related to PWA than other health professionals. The results also illustrate that healthcare professionals with moderate frequency of contact with PWA (1–20 times per week) present higher HPAQ scores compared to those with more frequent contact with PWA (>21 times per week) or with no contact at all. Last, though inconclusive evidence was presented, our data suggest that more years of work experience, especially when working in direct contact with PWA, can increase knowledge and skills.

## 4 Discussion

This study explored the knowledge and skills of Cypriot healthcare professionals when interacting with PWA in clinical settings. The findings highlighted several critical insights concerning the educational level of the healthcare professionals, differences between professions, professional experience, and frequency of interaction with PWA, and training needs.

Specifically, educational attainment was a significant predictor of HPAQ scores, with healthcare professionals holding a postgraduate degree outperforming those with an undergraduate degree. This aligns with the findings by Barrat (2018), which suggest that advanced education and practice skills equip healthcare professionals with deeper theoretical and practical knowledge, enabling them to implement effective communication to enhance patient outcomes. However, this study highlights the importance of making training accessible to all educational levels to ensure consistent standards of care across professions. By doing so, healthcare systems can bridge gaps and create opportunities for professional growth among healthcare professionals at varying levels. Ultimately, equitable access to education and training fosters a collaborative approach to care, benefiting both healthcare teams and patients.

Interestingly, although years of professional experience did not show significant differences in HPAQ scores, the regression analysis indicated a nuanced relationship influenced by interactions with other factors, such as the frequency of contact with PWA. These findings align with Nikkels et al. (2023), who noted that experience alone does not ensure competency unless combined with specific training. This proposes that the impact of professional experience is enhanced when it involves purposeful and meaningful interaction with PWA. Furthermore, this highlights the need for structured opportunities within healthcare settings that combine experiential learning with targeted professional development. In addition, the frequency of interaction with PWA emerged as a critical factor in shaping HPAQ scores, with healthcare professionals who worked with PWA at moderate frequencies reporting higher scores than those with no contact or intensive contact. These findings reflect the importance of consistent yet manageable exposure to PWA in fostering familiarity with communication strategies while avoiding the potential burnout associated with excessive caseloads (Carragher et al. 2024). Moderate interaction might provide healthcare professionals with the opportunity to develop their communication skills, reflect on their practice, and refine their approach over time. Thus, balanced exposure supports a sustainable model of professional engagement, ensuring high‐quality care of PWA while protecting the well‐being of healthcare practitioners.

Finally, as professional differences are concerned, SLTs reported significantly higher knowledge and skills compared to other healthcare professionals, including nurses, physicians, and rehabilitation experts (van Rijssen et al. 2022). This finding underscores the specialised training SLTs receive in communication disorders, highlighting the need for other healthcare professionals to receive targeted education and training in managing communication with PWA (Finch et al. 2017). Addressing this gap could facilitate better interdisciplinary collaboration, ensuring a more cohesive approach to PWA care and significantly improving patient outcomes.

Our findings align with and extend previous literature demonstrating that healthcare professionals often report limited confidence and knowledge when interacting with PWA, particularly in acute and high‐pressure clinical environments. Cameron et al. (2018) similarly identified variability in confidence levels across different professional groups, with speech‐language pathologists reporting greater confidence than other professionals, such as nurses or allied health staff. This echoes our results, which highlight disparities in perceived preparedness and frequency of communication with PWA across disciplines. Furthermore, van Rijssen et al. (2022) reported that professionals often feel underprepared and uncertain about how best to adapt their communication, particularly when time constraints or lack of training were factors, barriers also raised by participants in our study. Importantly, van Rijssen et al. also emphasised the need for tailored communication partner training (CPT) that is responsive to the specific needs and clinical contexts of different professional groups. Similarly, Finch et al. (2017) demonstrated that CPT can significantly improve speech‐language pathology students’ conversational abilities with PWA, suggesting that early and profession‐specific training may be crucial for developing communicative competence. Our findings reinforce this view and underscore that without structured opportunities for skill development, healthcare professionals, particularly those outside of speech‐language therapy, may continue to lack the skills and confidence needed to support communicative participation for PWA. The consistency across studies underlines the critical importance of embedding aphasia‐specific communication training in both undergraduate education and continuing professional development across healthcare disciplines.

## 5 Clinical Implications

The results highlight the need for structured communication training programs, such as Communication Partner Training, to address gaps in knowledge and skills among non‐SLT healthcare professionals in Cyprus. Our findings provide a foundation for both general communication training and the future implementation of CPT as a targeted, evidence‐based intervention tailored to the needs of specific professional groups. Communication Partner Training programs have been shown to improve healthcare professionals’ ability to support PWA by teaching compensatory communication strategies and fostering positive attitudes (Sullivan et al. 2024). Implementing Communication Partner Training in Cypriot healthcare settings can enhance the inclusivity of clinical environments, enabling PWA to participate more actively in healthcare decisions and rehabilitation planning (Hinckley and Jayes 2023).

Also, the findings emphasise the importance of tailoring training interventions to specific professional groups and educational levels. For example, while SLTs may benefit from advanced Communication Partner Training modules focusing on specific strategies, other healthcare professionals could benefit from introductory‐level training to build foundational knowledge and skills. This tiered approach aligns with recommendations from Bright and Reeves (2022), who advocate for scalable training solutions to address diverse healthcare needs. The above findings provide a foundation for future interventions aimed at improving the communication between healthcare professionals and PWA in Cyprus. Implementing structured communication training programs could significantly enhance the quality of care and support provided to PWA, ultimately improving patient outcomes and satisfaction with healthcare services (Burgener 2020).

### 5.1 Limitations

This study has limitations that should be acknowledged. First, the reliance on self‐reported data via the HPAQ may introduce response biases, as participants might overestimate or underestimate their competencies. Second, the relatively small sample sizes in some subgroups also limit the generalizability of findings. For example, the lack of significant differences in years of experience with PWA might be attributed to limited observations within certain interaction subgroups. Third, no face validity study was conducted to evaluate the appropriateness of the Greek version of the HPAQ for Cypriot‐Greek speakers. Since bilectal language context may have influenced interpretation, future research should include cultural and linguistic adaptation of the tool for the Cypriot setting. Finally, data on participants’ work status (e.g., full‐time vs. part‐time) or whether their roles were primarily clinical or managerial were not collected. These factors could have influenced the extent of their interactions with PWA and should be considered in future research.

### 5.2 Future Directions

Future research could incorporate objective assessments, such as observational studies, to validate self‐reported findings and aim for larger, more diverse samples to explore these interactions in greater detail.

## 6 Conclusion

This study provides a foundation for improving communication between healthcare professionals and PWA in Cypriot clinical settings. The findings highlight the need for targeted education and training initiatives, particularly for non‐SLT healthcare professionals, to address knowledge gaps and promote effective communication practices. Implementing Communication Partner Training programs can create more inclusive healthcare environments, enhance patient participation in decision‐making, and improve rehabilitation outcomes for PWA. By addressing these critical areas, Cypriot healthcare systems can take significant steps towards delivering equitable and high‐quality care for individuals with aphasia.

## Conflicts of Interest

The authors declare no conflicts of interest.

## Registration Number

This study received ethical approval: CNBC EP 2023.01.02

## Data Availability

The data generated during the current study and support the conclusions of this article are publicly available in the manuscript. Any further data queries and requests should be submitted to the corresponding author.
